# A Risk Signature With Inflammatory and T Immune Cells Infiltration in Colorectal Cancer Predicting Distant Metastases and Efficiency of Chemotherapy

**DOI:** 10.3389/fonc.2019.00704

**Published:** 2019-08-13

**Authors:** Xiang Hu, Ya-Qi Li, Xiao-ji Ma, Long Zhang, San-Jun Cai, Jun-Jie Peng

**Affiliations:** ^1^Department of Colorectal Surgery, Fudan University Shanghai Cancer Center, Shanghai, China; ^2^Department of Oncology, Shanghai Medical College, Fudan University, Shanghai, China

**Keywords:** tumor CEACAM8+ neutrophil, tumor-infiltrating lymphocytes, colorectal cancer, risk signature, survival analysis

## Abstract

In order to accurately predict oncological outcomes of colorectal cancer (CRC), we established a risk signature with tumor infiltrating neutrophils and T immune cells for prognosis. A total of 276 CRC patients from FUSCC, and 434 patients from TCGA cohort were enrolled in the study. A risk signature model in combination with CEACAM8+ neutrophils, CD3+, CD8+ T lymphocytes, and FOXP3+ regulatory T cells was established, and the relationships with patient clinicopathological characteristics and prognosis were evaluated. In TCGA cohort, high CEACAM8 expression was observed as an independent factor of poor disease-free survival (DFS), as well as inversely correlated with CD8 (*P* = 0.0035) and FOXP3 expression (*P* = 0.05). In the FUSCC cohort for validation, the association between CEACAM8+ neutrophils and DFS had been confirmed in CRC tissue (*P* = 0.026). Furthermore, a risk stratification was derived from integration of CEACAM8+ neutrophils and T immune cells. In both OS and DFS, the high-risk group all demonstrated worse prognosis than low-risk group, with statistical significance (all *P* < 0.001). In addition, the high-risk group was correlated with post-operative relapses with accurate prediction. Furthermore, the high-risk group identified a subgroup of CRC patients who appeared not to benefit from adjuvant chemotherapy. At last, predictive nomograms were constructed with recognized independent prognosticators, showing this risk signature increasing the predictive accuracy and efficiency for OS and DFS. In conclusion, incorporation of neutrophil into T lymphocytes could provide more accurate prognostic information in CRC, and this risk stratification predicted for survival benefit from post-operative chemotherapy.

## Introduction

Inflammatory reactions, always accompanied with immune response in tumor niches, play vital roles in tumorigenesis, cancer progression and resistance to therapy (1, 2). Tumor-infiltrating neutrophils (TIN) are reported as typically pro-tumor and are strongly associated with adverse prognosis in most human cancers (3). On the contrary, the relationships between neutrophils infiltration and prognostic outcomes generated heterogeneous results according to different tumor niches (4, 5). Indeed, there were different phenotypically distinct sub-population of neutrophils with conflicting functions, therefore high neutrophil plasticity was indicated under different tumor niches (6). Based on this plasticity, neutrophils showed different functions through polarization between an anti-tumoral (N1) and a pro-tumoral (N2) phenotype (7). So, in the present study, CEACAM8, as the great specificity and activated phenotype of neutrophils, was used to detect tumor-infiltrating neutrophils within colorectal cancers (CRC) (3). The preliminary aim was to assess the association of neutrophils to patient clinical and molecular characteristics, as well as prognosis. Furthermore, in order to accurately predict oncological outcomes of CRC, we wanted to establish predictive biomarkers of survival prognosis and treatment response, in addition of advanced surgical techniques, perioperative treatment, and adjuvant therapy. Although at present conventional TNM staging is the most frequently applied and well-recognized prognostic tool, but even patients with the same stage can have very distinct prognosis (8). As this staging system narrowly concentrated on the tumor cells without incorporating the effects of the host immune response. Hence, systematic analysis of tumor infiltrating neutrophils and immune cells in cancer tissue would thus enable us a precise classification in tumor progression. The major cell types in T lymphocytes comprised of CD3, CD8, and FOXP3 Tregs were included in this study. In the present study, we combined TIN and T immune cells in CRC and investigated their relation with clinical outcomes, especially in patients receiving adjuvant chemotherapy. At last, a risk signature model has been generated and provides a possible predictive system to evaluate outcomes for patients received adjuvant chemotherapy.

## Results

### Predictive Value of CEACAM8 in TCGA

**Figure 1:**
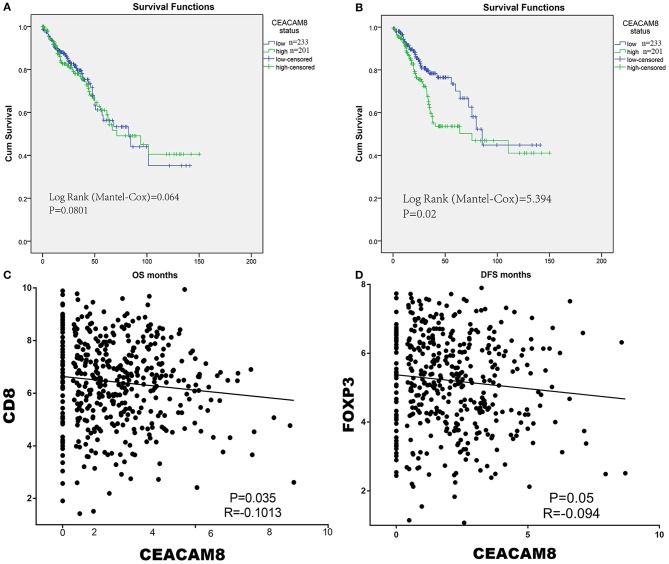
The overall survival curves were similar between high and low CEACAM8 groups **(A)**. On the contrary, Patients with high CEACAM8 had a significantly poorer disease-free survival than those with low level of CEACAM8 **(B)**. The pattern of CEACAM8 expression was negatively correlated with CD8 **(C)**, and negatively correlated with FOXP3 **(D)**.

In the discovery set (TCGA), we performed X-tile program to determine the cut-off values of CEACAM8 mRNA levels in tumor tissue, which were 1.92. Then patients with high and low CEACAM8 expression were identified for further analysis (≤1.92 as low CEACAM8 group and >1.92 as high CEACAM8 group). Kaplan–Meier survival analysis was carried out to compare overall survival (OS) according to CEACAM8. The overall survival curves were similar between high and low CEACAM8 groups by non-parametric with no statistically significant difference (log-rank test = 0.213, *P* = 0.645) (Figure 1A). On the contrary, Patients with high CEACAM8 had a significantly poorer disease-free survival (log-rank test = 5.364, *P* = 0.021, Figure 1B), than those with low level of CEACAM8. Using the online software “TIMER” to calculate the immune cell infiltration, we also performed those analysis. The same result was also achieved. See the Supplemental Figure 1. The ability of tumor-infiltrating neutrophils expressing CEACAM8 to regulate the immune-cell infiltration may account for this survival profit. To investigate the relationship of tumor-infiltrating neutrophils and immune cells, the relationships of CEACAM8 with CD3, CD8, and FOXP3 mRNA expression were assessed. The pattern of CEACAM8 expression was negatively correlated with CD8 (*r* = −0.1013, *P* = 0.035; Figure 1C), and negatively correlated with FOXP3 (*r* = −0.094, *P* = 0.05; Figure 1D). No significant correlation of CEACAM8 with CD3 (*r* = 0.16, *P* = 0.57) was seen.

### Association of CEACAM8+ TIN With Survival in FUSCC

**Figure 2:**
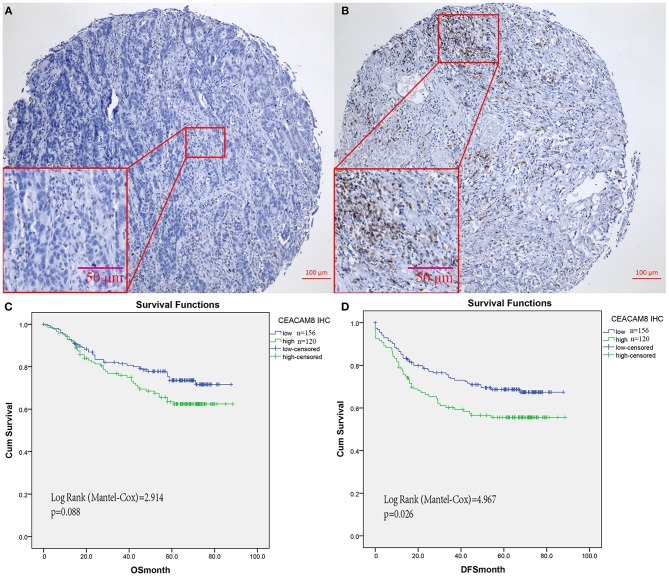
CEACAM8+ neutrophils in colorectal cancers from FUSCC for validation. Representative images of tissue cores as low **(A)**, or high **(B)**. Although TIN was not identified as an independent prognostic factor for overall survival **(C)**, Kaplan-Meier analysis showed that high TIN was significantly associated with worse DFS **(D)**.

In the FUSCC cohort for validation by IHC, based on the cutoff value of 17 in TIN (Figures 2A,B), Then patients with high and low TIN infiltration were identified for further analysis (≤17 per core as low TIN infiltration group and >17 as high infiltration group). Kaplan-Meier analysis showed that high TIN was associated with worse DFS of CRC patients (*P* = 0.026, Figure 2D). Although the presence of high TIN was not identified as an independent prognostic factor for overall survival (*P* = 0.088, Figure 2C). Not only CEACAM8+ cells, but also CD3+, CD8+, and FOXP3+ cells were observed in colorectal cancer tissues. To validate the relationship of tumor-infiltrating neutrophils and immune cells, the distributions of positively labeled cells with CEACAM8, CD3, CD8, and FOXP3 were assessed. The number of TIN expressing CEACAM8 was negatively correlated with CD8 cells (*r* = −0.123, *P* = 0.042), and no significant correlation of TIN with CD3+ and FOXP3+ cells (all *P* > 0.05) was observed.

**Figure 3:**
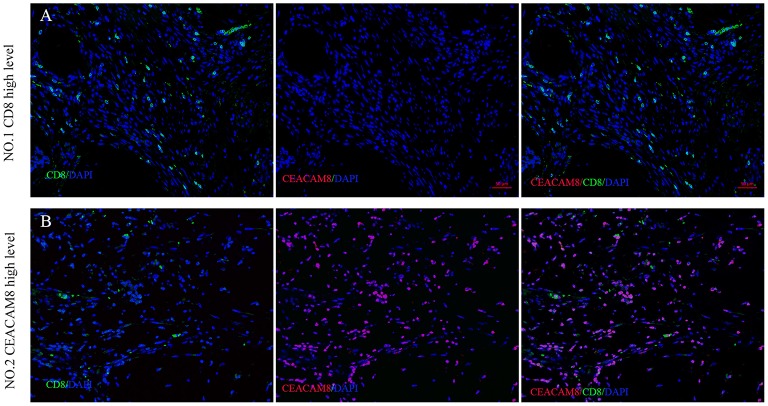
The association between CEACAM8+ neutrophils and CD8+ T cells in CRC. Representative image of colorectal cancer tissue in **(A)** high CD8+ T cells (green) with low density of CEACAM8+ (red), compared with the low CD8+ T cells (green) with high density of CEACAM8+ (red) **(B)**.

To validate the result that TIN was negatively linked with CD8+ cells in cancer tissue, multi-color immunofluorescence was performed using monoclonal antibodies recognizing CEACAM8, CD8, and DAPI. The number of CD8+ cells was significantly higher in tumors, when CEACAM8 expression decreased (Figure 3A). In addition, when TIN expressing CEACAM8 increased, the number of CD8+ cells was significantly lower (Figure 3B).

### Risk Stratification Based on CEACAM8+ TIN and T Immune Cells

**Figure 4:**
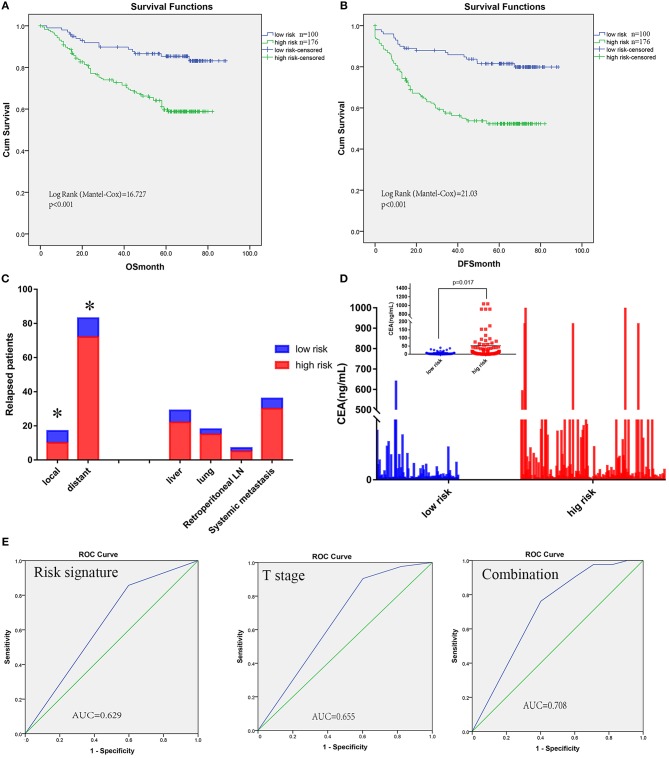
Risk signature model of tumor-infiltrating CEACAM8+ neutrophils plus immune cells and its prognostic value. Kaplan-Meier analysis showed high-risk signature was significantly associated with worse OS **(A)** and DFS **(B)**. The high-risk signature was correlated with distant recurrence significantly **(C)**. A significantly greater increase in CEA was observed in the high-risk signature group than the low-risk group **(D)**. The receiver operating characteristic (ROC) curves for predicting patient distant metastases using the risk signature, T stage or a combination of the two factors **(E)**. ^*^Significance compared between high and low-risk signature group.

To illustrate the correlation of tumor-infiltrating inflammatory and immune cells with clinicopathological factors and prognosis, we constructed a risk signature model predicting survival based on TIN and immune cells. We divided the patients into high and low-risk groups based on the four immune cells with an NTP algorithm to assess the prognostic impact of immune profile in CRC patients. What is more, we transposed the risk signature into a predictive score to make the model easier to use in further study. The high-score group determined by predictive score >2 had been identified as the high-risk group, and the low-risk group with the score ≤2 (Table 1). Figure 4A showed the worse survival of OS in the high-risk group based on predictive score as described above (log-rank test = 16.727, *P* < 0.001). Regarding disease-free survival, the high-risk group also demonstrated worse DFS than low-risk group, with statistical significance (Figure 4B, log-rank test = 21.03, *P* < 0.001).

**Table 1:** Baseline characteristics in patients with high and low-risk signatures.

**Variables, *N* (%)**	**Inflammation risk signature**		***P*-value**
	**Low risk (*n* = 100)**	**High risk (*n* = 176)**	
Gender			
Male	61 (61.0)	103 (58.5)	0.687
Female	39 (39.0)	73 (41.5)	
Age, years	56.6 ± 11.7	57.52 ± 10.6	0.28
TNM stage			0.006
I	11 (11.0)	10 (5.7)	
II	34 (34.0)	47 (26.7)	
III	49 (49.0)	83 (47.2)	
IV	6 (6.0)	36 (20.5)	
T stage			0.008
T2	21 (21.0)	22 (12.5)	
T3	26 (26.0)	28 (15.9)	
T4	53 (53.0)	126 (71.6)	
N stage			0.149
N0	52 (52.0)	68 (38.6)	
N1	27 (27.0)	55 (31.3)	
N2	21 (21.0)	52 (29.5)	
M stage			0.001
M0	94 (94.0)	140 (79.5)	
M1	6 (6.0)	36 (20.5)	
Grade			0.854
Well/ moderate	78 (78.0)	142 (80.7)	
poor	22 (22.0)	34 (19.3)	
Histological type			0.81
Adenocarcinoma	95 (95.0)	166 (94.3)	
Mucinous	5 (5.0)	10 (5.7)	
Lymph node examined			0.448
Median	15 ± 6	14 ± 5	
Perineural invasion			0.565
Negative	82 (82.0)	149 (84.7)	
Positive	18 (18.0)	27 (15.3)	
Vascular invasion			0.297
Negative	72 (72.0)	116 (65.9)	
Positive	28 (28.0)	60 (34.1)	
Adjuvant Chemotherapy			0.222
No	19 (19.0)	30 (17.0)	
Yes	72 (72.0)	117 (66.5)	
MS status/MMR status			0.561
MSS/MMR-proficient	66 (66.0)	110 (62.5)	
MSI/MMR-deficient	34 (34.0)	66 (37.5)	
CEA (ng/ml)	8.687 ± 3.276	52.77 ± 14.54	0.017

*MMR, mismatch repair; MS, microsatellite; MSS, microsatellite stability; MSI, microsatellite instability*.

### Risk Stratification With CEACAM8+ TIN and T Immune Cells Is an Independent Predictor of Both OS and DFS

Univariate and multivariate analyses for OS and DFS were carried out (Tables 2, 3). Univariate analysis for OS revealed that high-risk signature, advanced TNM stage, T4 stage, presence of lymph node metastasis, distant metastasis, vascular invasion and high CEA (>10 ng/ml) were significantly associated with poor OS. Multivariate analysis balancing those factors showed that high-risk signature (*HR* = 2, *P* = 0.02), advanced TNM stage (HR = 4.468, *P* < 0.001), and T4 stage (*HR* = 1.704, *P* = 0.0036) were independent predictors of poor OS. Regarding disease-free survival, univariate analysis found that high-risk signature, advanced TNM stage, T4 stage, presence of lymph node metastasis, distant metastasis, vascular invasion, perineural invasion, retrieved lymph nodes (>12) and high CEA (>10 ng/ml) were significantly associated with poor DFS. High-risk signature (*HR* = 2.361, *P* = 0.01), advanced TNM stage (*HR* = 4.258, *P* < 0.001), and T4 stage (*HR* = 1.516, *P* = 0.0045) were independent predictors of poor DFS in multivariate analysis.

**Table 2:** Univariate and multivariate analysis for overall survival.

**Variables**	**Univariate analysis**			**Multivariate analysis**		
	**Hazard ratio**	**95% CI**	***P*-value**	**Hazard ratio**	**95% CI**	***P*-value**
High-risk signature	3.039	1.733–5.328	<0.001	2	1.113–3.599	0.02
Male gender	1.255	0.797–1.976	0.328			
Age >70	1.581	0.915–2.734	0.101			
TNM stage IV	5.118	3.574–7.330	<0.001	4.468	1.883–10.603	0.001
T stage, T4	10.531	2.580–42.981	0.001	1.704	1.035–2.805	0.036
N stage, N2	4.139	2.332–7.346	<0.001	0.778	0.532–1.138	0.196
M1 stage	9.06	5.735–14.314	<0.001			
Grade, poor	1.36	0.880–2.103	0.166	0.931	0.3–2.891	0.901
Lymph node examined	0.72	0.465–1.114	0.14			
Mucinous Adenocarcinoma	0.865	0.316–2.364	0.77			
Perineural invasion	1.639	0.980–2.740	0.06			
Vascular invasion	2.44	1.578–3.776	<0.001	1.22	0.721–2.066	0.459
Adjuvant therapy	0.67	0.315–1.425	0.299			
MSI	0.923	0.589–1.444	0.725			
CEA >10ng/ml	2.705	1.713–4.273	<0.001	0.963	0.557–1.664	0.892

*MMR, mismatch repair; MS, microsatellite; MSS, microsatellite stability; MSI, microsatellite instability; LN, lymph nodes*.

**Table 3:** Univariate and Multivariate analyses of prognostic factors for disease-free survival.

**Variables**	**Univariate analysis**			**Multivariate analysis**		
	**Hazard ratio**	**95% CI**	***P*-value**	**Hazard ratio**	**95% CI**	***P*-value**
High-risk signature	3.033	1.838–5.004	<0.001	2.361	1.408–3.957	0.01
Male gender	1.322	0.883–2.009	0.172			
Age >70	1.214	0.711–2.072	0.478			
TNM stage IV	5.363	3.837–7.496	<0.001	4.258	2.037–8.897	<0.001
T stage, T4	2.401	1.630–3.537	0.001	1.516	1.010–2.277	0.0045
N stage, N2	1.886	1.498–2.374	<0.001	0.783	0.562–1.092	0.15
M1 stage	10.069	6.567–15.439	<0.001			
Grade, poor	1.168	0.788–1.730	0.439	1.156	0.433–3.087	0.772
LN examined >12	0.662	0.447–0.981	0.04	0.702	0.466–1.057	0.09
Mucinous Adenocarcinoma	0.872	0.355–2.144	0.765			
Perineural invasion	1.892	1.203–2.977	0.006	1.419	0.88–2.289	0.151
Vascular invasion	2.223	1.500–3.296	<0.001	0.876	0.539–1.422	0.591
Adjuvant therapy	0.568	0.281–1.147	0.114			
MSI	0.946	0.631–1.416	0.786			
CEA >10 ng/ml	3.318	2.211–4.981	<0.001	1.375	0.857–2.204	0.187

*MMR, mismatch repair; MS, microsatellite; MSS, microsatellite stability; MSI, microsatellite instability; LN, lymph nodes*.

### Risk Stratification of CEACAM8+ TIN and T Immune Cells Is Correlated With Distant Metastases

To address why a high-risk signature had a correlation with poor prognosis, relapse patterns were deeply determined. All patients with relapse could be properly assessed for this analysis. Relapse involving anastomosis and pelvic lymph nodes were defined as local; Relapse involving organs such as the liver, lungs, peritoneum, and retroperitoneal nodes were defined as distant. The high-risk signature was found to correlate with distant recurrence significantly (Figure 4C). CEA levels, one of the most widely used tumor markers for detecting recurrence in colorectal cancer, were included in this comparative analysis. A significantly greater increase in CEA was observed in the high-risk signature group than that in the low-risk signature group (Figure 4D).

### Extension of the Distant Metastases Prognostic Model With Risk Signature

To improve the prognostic accuracy for current TNM staging system, we established a predictive model for CRC patients by combining T stage and this risk signature with TIN plus T immune cells. The area under the curve (AUC) as prediction based on the T staging (0.655) was comparable with that for the risk signature with TINs plus T immune cells (0.629), and the combination of both factors achieved the highest AUC value (0.708), see Figure 4E.

### Predictive Value of Risk Signature for Adjuvant Chemotherapy Benefit

**Figure 5:**
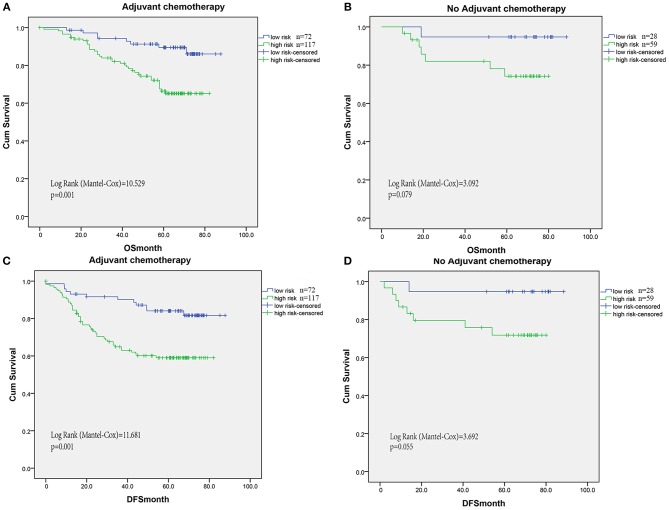
For patients without adjuvant chemotherapy treatment, the risk signature was not significantly associated with OS **(B)** and DFS **(D)**. However, high risk signature patients had significantly shorter OS **(A)** and DFS **(C)** in patients with adjuvant chemotherapy (log-rank test = 10.529, *P* < 0.001, **A**).

Increased levels of inflammation cells have been reported to promote tumor invasion and reduce chemotherapy efficacy and immunologic death. Thus, we evaluated the benefit of fluorouracil-based adjuvant chemotherapy according to the risk signature model of TIN plus T immune cells. For patients without adjuvant chemotherapy treatment, the risk signature was not significantly associated with OS and DFS (Figures 5B,D). However, high risk signature patients had significantly shorter OS in patients with adjuvant chemotherapy (log-rank test = 10.529, *P* < 0.001, Figure 5A). Similarly, the association between risk signature and DFS was also significant in patients with adjuvant chemotherapy (log-rank test = 11.681, *P* = 0.001, Figure 5C).

### Extension of Risk Signature With Current Prognostic Models

**Figure 6:**
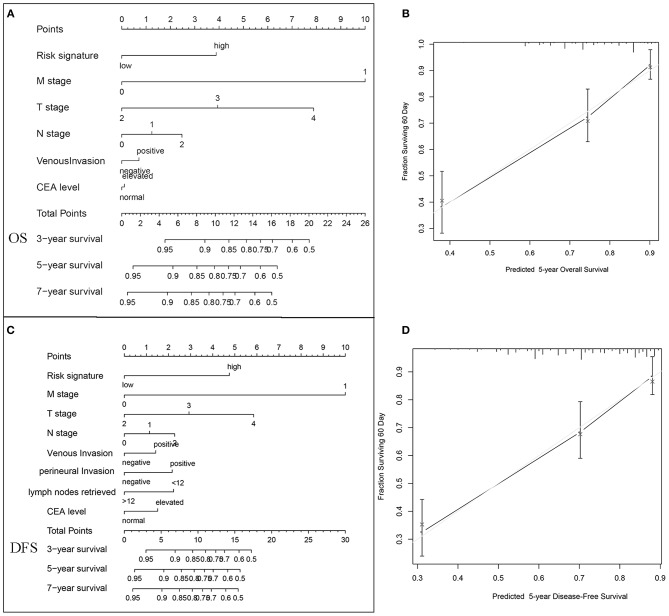
Nomogram, and calibration plots for the prediction of OS and DFS in colorectal cancers. Nomograms for OS **(A)** and DFS **(C)** based on this risk signature and other well-recognized prognosticators. The calibration of nomograms for both OS **(B)** and DFS **(D)**, demonstrating a great prediction accuracy of this risk signature nomograms.

To visualize the prognostic application of this risk signature, we then created nomograms for OS and DFS based on this risk signature and other well-recognized prognosticators (Figures 6A,C, respectively). Figures 6B–D showed the calibration of nomograms for both OS and DFS, demonstrating a great prediction accuracy of this risk signature nomograms. What is more, after incorporating with TNM staging model, this risk signature could increase the predictive accuracy and efficiency for both OS and DFS. The combination with this risk signature had higher c-indices (0.793) than TNM staging alone (0.769).

## Discussion

Tumor infiltration of inflammatory cells and T lymphocytes was frequently observed in CRC, and there were several previous studies finding the infiltration of both inflammatory cells and T lymphocytes were linked to different prognosis (9, 10). In this study, we have investigated the survival impact of CEACAM8-positive neutrophils in CRC and illustrated the correlation of tumor-infiltrating inflammatory and immune cells with prognosis. We found that the CEACAM8^+^ neutrophils could be used to predict survival in CRC. Furthermore, we generated a risk signature model, which was defined by infiltration of inflammatory and immune cells, predicting prognosis independent of TNM staging. The high-risk signature was not only associated with tumor progression, it could also predict the efficiency of chemotherapy.

Neutrophils, a canonical leucocyte, have been considered as one of first-line defenders against infectious microorganisms. At the meantime, neutrophils infiltrating tumor tissues play vital roles in tumor development and progression (11). In the early start of colorectal tumorigenesis, infiltration of neutrophils has been involved, as neutrophils in colorectal adenomas were increased significantly compared to adjacent normal mucosa and correlated with adenoma size (11). N-nitrosamines generated from neutrophils may be responsible, as they are known to be carcinogenic (12–14). In addition, an hMSH2-dependent G2/M checkpoint arrest could be induced by activated neutrophils, then replication errors occurred in colon epithelial cells (15). These findings are consistent with our observations, indicating neutrophils infiltration as an adverse prognostic factor in adenoma-carcinoma sequence and cancer progression. On the contrary, the relationships between neutrophils infiltration and prognostic outcomes generated heterogeneous results according to different tumor types. For example, in gastric cancer, presence of neutrophils was a favorable factor for disease-free survival (4), whereas also in gastric cancer, poor outcomes were followed with neutrophils infiltration in another study (16). This inconsistency may result from the methods used in assessing neutrophil infiltration. Hematoxylin-eosin staining alone or CD15 cell marker staining for neutrophils were utilized in varied studies. CD15, which was not exclusively expressed in neutrophils, also were observed on eosinophils and part of monocytes. Apart from this, some tumor cells were reported to express CD15 (17, 18). In light of the specificity, we decided to use CEACAM8 to identify neutrophils (19). So CEACAM8-positive TINs were observed up-regulated in tumor tissues relative to match normal tissues. In addition, the inconsistent results for oncological outcome obtained in colorectal cancer (20, 21) may narrowly concentrated on the inflammation without taking the effects of the host immune response into consideration. Hence, integration of tumor infiltrating neutrophils and immune cells in cancer tissue would thus enable us a precise classification in tumor progression. And infiltrating neutrophils may depend on tumor location and MSI status, in which the microenvironment dependent function probably reflected the tailored immune milieu and contexts, while this background data were missing in those studies. Above all, in these two cohorts with balanced baseline characteristic CEACAM8 low expression gained a DFS benefit compared with high expression.

Neutrophil cells have been paid on with most attention in the pathogenesis and progression of cancers, due to their immune destruction evading and tumor-promotion capability. Although neutrophils infiltration could be recognized as an independent prognostic factor in this study, whereas narrowly DFS was correlated and inconsistency also arose. It was responsible not to incorporate the effects of the host immune response. Hence, incorporation of TIN into T immune cells could provide more accurate prognostic information for the risk stratification of CRC. We had established a risk signature model with four types of cells (TIN, CD3+, CD8+ T cells, and FOXP3+ cells), and this model showed nice correlation with patients' prognosis (DFS: *HR* = 2.361, *P* = 0.01; OS: *HR* = 2, *P* = 0.02). This risk signature model including inflammatory cells and T lymphocytes showed better correlation with survival prognosis than neutrophil infiltration alone. The number of TIN was significantly correlated with that of CD8+ T cells, suggesting that those cells cooperate to induce inflammation affecting cancer invasion, although there were not apparent associations in TIN with CD3+ and FOXP3+ cells. So, we believed that neutrophil cells somehow moderated immune cell status relative to tumor progression.

Besides the clinical relevance of the risk-signature to survival prognosis, the risk signature was also correlated with post-operative relapse. The mechanisms behind the clinical relevance of the risk-signature to recurrence pattern may mainly lie in that tumor-induced alteration of neutrophils and T lymphocytes acted to produce premetastatic niches then promote distant metastasis (2, 22, 23). Exactly, neutrophil cells in cancer niches were able to inhibit anti-tumor T-cells such as CD8 cells. As expected, this high-risk signature represented a more aggressive phenotype in the cohort, accompanied advanced TNM staging as well as another prognostic factor: high CEA value. Furthermore, to identify more clinical implications of this risk signature additional of its survival prognosis, we also carefully assessed the relation between this risk signature and chemotherapy. We found that among patients with chemotherapy, those with low risk signature were easier to have longer OS and DFS compared with those with high risk signature, while there was no correlation in patients without chemotherapy. These indicated that this risk signature could be an important factor for predicting the efficiency of chemotherapy. In line with our observation, inflammatory and immune cells were reported be responsible in patients most likely to benefit from chemotherapy. As in HCC, CCL2, and CCL17 expressed by TIN recruited macrophages and Treg cells promoting neovascularization and resistance to anti-angiogenesis therapy (24). Additionally, interferonγ derived from CD8+ T cell reversed stroma-mediated chemo-resistance in the tumor niches (25). The increased proinflammatory cytokines, including IL-6 and IL-8 levels, were a part of explanations for multidrug and apoptosis resistance in cancers (26, 27). Therefore, the roles of tumor-infiltrating neutrophil and T immune cells were becoming clear more and more, and indicated that this risk signature based on these cells might be utilized to stratify patients by tumor niche status and immune cell profile.

Several additional limitations are important to consider in interpreting the results of the present study. There existed some issues to be addressed in the future work. (1) This study, as its retrospective design and the relatively small sample size, was not enough for validation of the outcome. (2) Although we identified the impact of the interaction of TIN and T immune cells involved in tumor progression, the underlying mechanisms through which those major immune cells crosstalk with each other remains unrevealed. (3) Prognostic serum marker for risk-signature will be easier to use in bedside, which are our next concern and under investigation.

In conclusion, CEACAM8^+^ neutrophils could be used to predict survival in CRC. Incorporation of neutrophils into T lymphocytes could provide more accurate prognostic information for the risk stratification. So, this risk signature model combined with those cells has been generated and showed nice correlation with patients' prognosis.

## Materials and Methods

### Study Population

This study included two independent cohorts of CRC patients. The discovery cohort enrolling 434 CRC was obtained from the cancer genome atlas (TCGA) database available from Cancer Genomics Browser of University of California Santa Cruz (https://genome-cancer.ucsc.edu/). Detailed CEACAM8, CD3, CD8, and FOXP3 expression data were chosen from the TCGA database defined previously (28). The validation set enrolling 276 CRC patients was obtained from Fudan University Shanghai Cancer Center (FUSCC) between 2007 and 2009. Only patients with fully characterized tumors, intact overall survival (OS) and disease-free survival (DFS), information were included. All patient data are collected, including age, race, tumor location, year of diagnosis, primary tumor size, histological grade, number of lymph nodes examined, type of radiation, marital status, preoperative multimodal treatment, details of the surgical procedure, occurrence of complications, post-operative histopathology, application of adjuvant therapy, and follow-up (date of last visit, tumor recurrence, date of tumor-related or unrelated death, overall survival, and disease-free survival). All patients from FUSCC dataset have provided written informed consent. The research protocol was reviewed and approved by the institutional review board of the FUSCC.

### Tissue Microarray (TMA) Construction and Immunohistochemistry (IHC) Staining

The TMA used for this study includes 276 unselected, non-consecutive, primary, and sporadic colorectal cancers enrolled between January 2007 and November 2009 in FUSCC. Construction of this TMA has been previously described in detail (29). IHC were performed with appropriate antibodies (anti-CEACAM8 antibody, BD Biosciences; anti-CD3e, CD8a, FOXP3 from Cell Signaling Technology). The number of positive cells per field was estimated using Image Pro plus 6.0 (Media Cybernetics Inc., Bethesda, MD). The immunostaining was evaluated by two pathologists blinded to the clinical information. Discordances were resolved by re-review to consensus or the third reviewer.

The cutoff values for survival analysis was determined by X-tile 3.6.1 software (30) (Yale University, New Haven, CT, USA). In detail, X-tile plots provided a single, global assessment of every possible way of dividing a population into low-, and high-level marker expression. Different cut-points were presented in a right triangular grid. The intensity of the color of each cutoff point represented the strength of the association. What is more, The X-tile software provided an “on-the-fly” histogram of the resulting population subsets along with an associated Kaplan-Meier curve.

### Prognostic Prediction

The risk signature model was generated by combining expression pattern of CEACAM8, CD3, CD8, and FOXP3 by using Nearest Template Prediction (NTP) algorithm, in which a model of risk stratification is made as implemented in the NTP module of the GenePattern analysis toolkit (http://software.broadinstitute.org/cancer/software/genepattern/). Neutrophil infiltration was regarded as cells downregulating host immune response to cancer, while CD3, CD8, and FOXP3 T cells as cells immunoactivities. Hence, the predictive score was summed by following scores: (1) high CEACAM8 defined as 1 point; (2) low CEACAM8 defined as 0 point; (3) high CD3, CD8, and FOXP3 as 0 point, and (4) low CD3, CD8, and FOXP3 as 1 point. The final cutoff value was defined as two points in total.

### Statistical Analysis

Statistical evaluation was performed using IBM SPSS statistics Version 22 (SPSS Inc; IBM Corporation Software Group, Somers, NY). The Chi-square test or Fisher exact test was utilized for exploratory comparisons of patient groups. All statistical tests were performed 2-sided, and *P* < 0.05 were considered to be statistically significant. OS and DFS were estimated with the Kaplan-Meier method, and the log-rank (Mantel-Cox) test was used to compare independent subgroups. Cox proportional hazard models were used to investigate the effect on survival of multivariable relationships among covariates including the age at diagnosis, gender, stage at diagnosis, histological type, histological grade and treatment. Stage, status of perineural, and vascular invasion or any known clinical characteristics supposed to affect the prognosis were the stratified variable. Hazard ratios (HRs) and 95% confidence intervals (CIs) for multivariate analyses were computed using the Cox proportional hazards regression models. The R software version 3.4.3 and the “rms” package (R Foundation for Statistical Computing) were applied to perform the nomogram analysis and calibration plot.

## Ethics Statement

This study was carried out in accordance with the recommendations of the ethics committee of FUSCC and approval from the institutional review board. All patients gave written informed consent in accordance with the Declaration of Helsinki.

## Author Contributions

XH, J-JP, and S-JC conceived and designed the experiments. XH, Y-QL, S-JC, and LZ analyzed the data. XH and XM contributed the reagents, materials, analysis tools. XH wrote the paper.

### Conflict of Interest Statement

The authors declare that the research was conducted in the absence of any commercial or financial relationships that could be construed as a potential conflict of interest.

## Supplementary Material

Supplemental Figure 1 Patients with high CEACAM8 had a significantly poorer survival than those with low level of CEACAM8 in online software “TIMER”.
