# Supplementary figures and images for: A Risk Signature With Inflammatory and T Immune Cells Infiltration in Colorectal Cancer Predicting Distant Metastases and Efficiency of Chemotherapy
Source: Front Oncol. 2019 Aug 13;9:704. doi: 10.3389/fonc.2019.00704 (PMC6700227; doi:10.3389/fonc.2019.00704)

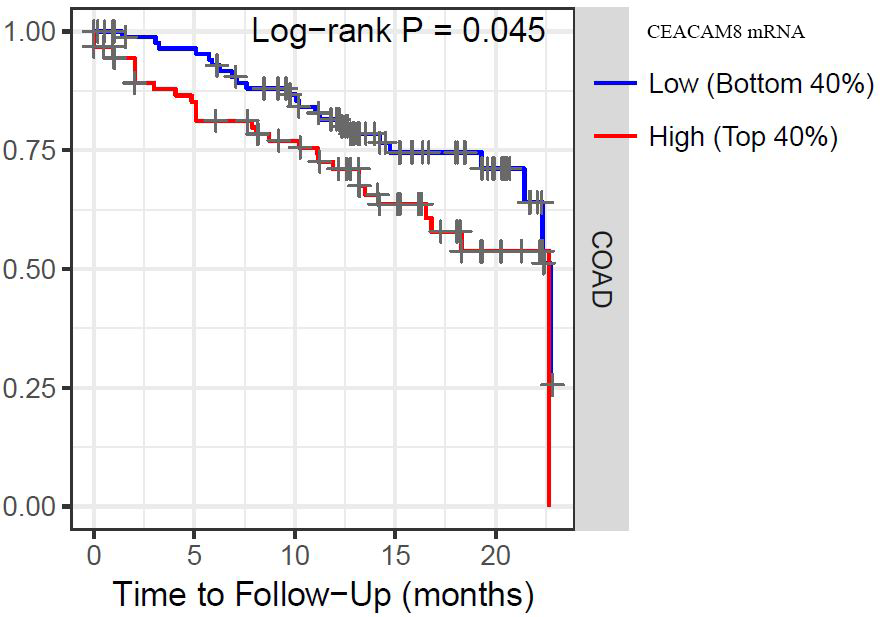

Supplement: Supplemental Figure 1 — Patients with high CEACAM8 had a significantly poorer survival than those with low level of CEACAM8 in online software “TIMER”. [file Image_1.TIF]
